# δ-secretase in neurodegenerative diseases: mechanisms, regulators and therapeutic opportunities

**DOI:** 10.1186/s40035-019-0179-3

**Published:** 2020-01-06

**Authors:** Zhentao Zhang, Ye Tian, Keqiang Ye

**Affiliations:** 1 Department of Neurology, Renmin Hospital of Wuhan University, Wuhan, 430060 People’s Republic of China; 2 Department of Pathology and Laboratory Medicine, Emory University School of Medicine, Atlanta, GA 30322 USA

**Keywords:** AEP, Alzheimer’s disease, Parkinson’s disease, C/EBPβ, TrkB, TDP-43

## Abstract

Mammalian asparagine endopeptidase (AEP) is a cysteine protease that cleaves its protein substrates on the C-terminal side of asparagine residues. Converging lines of evidence indicate that AEP may be involved in the pathogenesis of several neurological diseases, including Alzheimer’s disease, Parkinson’s disease, and frontotemporal dementia. AEP is activated in the aging brain, cleaves amyloid precursor protein (APP) and promotes the production of amyloid-β (Aβ). We renamed AEP to δ-secretase to emphasize its role in APP fragmentation and Aβ production. AEP also cleaves other substrates, such as tau, α-synuclein, SET, and TAR DNA-binding protein 43, generating neurotoxic fragments and disturbing their physiological functions. The activity of δ-secretase is tightly regulated at both the transcriptional and posttranslational levels. Here, we review the recent advances in the role of δ-secretase in neurodegenerative diseases, with a focus on its biochemical properties and the transcriptional and posttranslational regulation of its activity, and discuss the clinical implications of δ-secretase as a diagnostic biomarker and therapeutic target for neurodegenerative diseases.

## Background

Neurodegenerative diseases are a group of disorders that are characterized by the progressive degeneration of neurons in the central or peripheral nervous system. The most common neurodegenerative diseases include Alzheimer’s disease (AD), Parkinson’s disease (PD), frontotemporal dementia (FTD), amyotrophic lateral sclerosis (ALS), etc. The prevalence of neurodegenerative diseases is increasing, partly owing to the extensions in lifespan, but effective treatments are still lacking. These diseases are characterized by the aberrant aggregation of pathological proteins and selective neuronal damage in the nervous system, but the molecular mechanisms of the pathogenesis remain elusive [1, 2]. In 2008, we reported that a cysteine protease, asparagine endopeptidase (AEP), cleaves SET, a DNase inhibitor, and mediates neuronal cell death [3]. Since then, accumulating evidence suggests that AEP is activated in the aged brain and participates in the aggregation of pathological proteins and neuronal damage. It has been suggested that AEP may play a role in the onset and progression of AD, PD, FTD and ALS. This review summarizes the current knowledge on the roles of AEP in neurodegenerative diseases.

## Main text

### AEP structure and biochemical features

Mammalian AEP is a cysteine protease that specifically cleaves its substrates after asparagine residues. It is classified as a member of clan CD and family C13 of cysteine proteases (EC 3.4.22.34). The activity of AEP has been identified in plants, mammals, *Blastocystis*, and trematodes, indicating that it plays a fundamental role in different species [4–7]. The plant AEP was named vacuolar processing enzyme (VPE), since it has a strict specificity for cleaving after asparagine residues and exerts its effect on plant vacuoles [5, 8, 9]. The plant AEP is believed to be a key executioner involved in controlling plant programmed cell death [10]. In humans, AEP is encoded by the LGMN gene located on chromosome 14q32.12. Similar to other enzymes, such as caspases and cathepsins, AEP is synthesized as an inactive proenzyme that contains C- and N-terminal propeptides [4, 11, 12]. Crystal structures reveal that the C-terminal prodomain covers the caspase-like AEP enzyme core and prohibits the access of substrate to the enzyme core, thus blocking its enzymatic activity [13, 14]. Under acidic pH, the N- and C-terminal propeptides are erased through autocatalysis, activating AEP. Thus, the enzymatic activity of AEP is tightly regulated by pH [11, 12]. In addition to its asparaginyl endopeptidase activity, AEP has also been reported to exert carboxypeptidase and peptide ligase activity [4, 14–16]. However, the physiological and pathological effects of carboxypeptidase and peptide ligase activity remain unknown.

### The physiological functions of AEP

AEP is most abundant in the kidney, placenta, spleen, liver, testis, and thymus [17]. In the kidney, AEP is most abundantly expressed in proximal tubular cells (PTCs) and controls extracellular matrix remodeling through the degradation of fibronectin [18]. Mice lacking AEP accumulate a discrete set of proteins in the endosomes and lysosomes of their kidney PTCs, suggesting that AEP plays a role in the normal catabolism of proteins in PTCs [19]. AEP knockout mice develop proteinuria and a decreased glomerular filtration rate, indicating that AEP is required for normal kidney functions [19]. In addition, AEP is also implicated in the immune response. To induce an efficient immune response, foreign protein antigens must be processed by peptidases to generate peptides for presentation to T cells. It has been reported that AEP processes the microbial antigen tetanus toxin C fragment (TTCF) for major histocompatibility complex (MHC) class II presentation in vitro [20]. However, a subsequent study found that T cells and antibody responses to TTCF were normal in AEP-deficient mice, suggesting that other proteases can substitute AEP to enable antigen processes in the absence of AEP [21]. AEP may also completely digest the potential antigen and destroy antigen presentation, inducing immune tolerance [22]. Furthermore, AEP is also involved in the cleavage of the invariant chain chaperone of MHCII [23].

Mice lacking AEP develop fever, cytopenia, hepatosplenomegaly, and hemophagocytosis. Extramedullary hematopoiesis was observed in the spleen of AEP knockout mice, indicating that AEP plays a pivotal role in the development of blood cells [24]. Furthermore, AEP was found to regulate the activity of osteoclasts and bone resorption [25]. Interestingly, the osteoclast inhibitory activity of AEP was mapped to its C-terminal fragment and is independent of its protease activity [26]. Despite advances in the understanding of the physiological role of AEP, its role in the central nervous system has not been illustrated.

### Role of AEP in Alzheimer’s disease

AD is the most common neurodegenerative disease and is characterized by the accumulation of amyloid-β (Aβ) along with aggregated forms of the microtubule-associated protein tau within the brain. These events finally drive the clinical expression of dementia. The pathophysiology of AD is not yet fully understood, but Aβ has been proposed to be the initiator of a complex pathogenic cascade that causes AD. This amyloid cascade hypothesis has been the predominant framework in the past two decades in the AD research field [27–29]. Furthermore, tau was found to mediate Aβ-induced neurodegeneration and plays a key role in AD [30, 31]. Substantial efforts have been made to develop treatments targeting Aβ and tau. Unfortunately, they have thus far failed to produce effects that are clinically significant [32–35]. There is a growing consensus that gaining a better understanding of the underlying molecular mechanisms and investigating the role of novel etiopathological factors is urgently needed. We have identified that AEP is a novel protease that regulates both Aβ and tau pathology in AD.

#### AEP cleaves APP and promotes Aβ production

Aβ is produced by the sequential proteolytic cleavage of the transmembrane protein amyloid precursor protein (APP) by β- and γ-secretase. β-Secretase cleavage of APP generates the C-terminal C99 fragment, which is further cleaved by γ-secretase and generates Aβ. Alternatively, APP can be cleaved by α-secretase within the Aβ sequence and preclude the generation of Aβ [36]. Except for α-, β-, and γ-secretase, other proteases have also been identified to cleave APP and regulate its physiological and pathological functions [37, 38]. For example, it has been reported that η-secretase processing of APP generates fragments that inhibit neuronal activity in the hippocampus [38]. We identified that AEP cleaves APP after N585, yielding the APP (586–695) fragment, which is readily cleaved by β- and γ-secretase and generates Aβ, supporting that AEP possesses secretase activity. Therefore, we renamed AEP δ-secretase [39]. In addition to N585, δ-secretase also cleaves APP after N373, producing the APP (1–373) fragment, which is secreted into the extracellular space and is toxic to neurons. The knockout of δ-secretase in the APP/PS1 and 5XFAD mouse models substantially alleviated the deposition of Aβ and the pathophysiological changes in the brain [39]. These results indicate that δ-secretase regulates Aβ pathology in mouse models of AD.

#### δ-Secretase promotes tau pathology in AD

In addition to Aβ, the aggregation of phosphorylated and fragmented tau is another pathological hallmark of AD. It has been reported that the proteolytic processing of tau regulates its aggregation and neurotoxic effects. Several proteases have been found to mediate the fragmentation of tau, such as caspses [40–42], calpains [43, 44], and thrombin [45]. We identified a tau fragment, tau (1–368), in the brains of AD patients and found that δ-secretase was responsible for the cleavage of tau and the generation of this fragment. Both *in vitro* and in vivo data indicate that δ-secretase cleaves tau after N368, yielding a tau (1–368) fragment. Compared to full-length tau, this fragment is more prone to phosphorylation. The cleavage of tau by δ-secretase disturbed its microtubule assembly activity [46]. Furthermore, the tau (1–368) fragment binds to TrkB, the cognate receptor of brain-derived neurotrophic factor (BDNF), and blocks neurotrophic signals, inducing neuronal cell death [47]. Furthermore, the activity of δ-secretase and the cleavage of tau are increased in an age-dependent manner [46]. Thus, δ-secretase may play a key role in AD pathogenesis. Indeed, the deletion of δ-secretase from tau P301S transgenic mice partially reversed the neuropathological and electrophysiological changes in the mice [46].

In addition to APP and tau, δ-secretase also cleaves SRPK2, which plays an important role in RNA splicing by phosphorylating SR-splicing factors [48]. The cleavage of SRPK2 by δ-secretase increases its nuclear translocation as well as kinase activity. This event augments exon 10 inclusion during tau mRNA splicing and induces the imbalance between the expression of 4R-tau and 3R-tau, promoting tau aggregation in tauopathy [49]. These results support the pivotal role of δ-secretase in regulating tau pathology.

δ-Secretase is the only reported age-dependent protease that simultaneously regulates both APP and tau pathology in AD [39, 46]. We further investigated whether δ-secretase-derived APP (586–695) and tau (1–368) fragments are sufficient to trigger AD-like pathology. We found that the δ-secretase-derived fragments, APP (586–695) and tau (1–368), additively drive AD pathogenesis and cognitive dysfunction. Remarkably, the tau (1–368) fragment binds and activates the transcriptional factor STAT1, which further upregulates BACE1 transcription and Aβ production. Strikingly, Aβ in turn promotes δ-secretase expression, forming a vicious circle that mediates the progression of AD pathology. Thus, δ-secretase-cleaved tau and APP fragments synergically promote the onset and progression of AD [50]. This notion amends the Aβ cascade hypothesis by illustrating that tau pathology not only is downstream of Aβ but also drives Aβ pathology. Considering the synergic effect of δ-secretase-derived tau and APP fragments, targeting this protease will provide an unprecedented advantage over the strategy targeting either APP or tau alone.

#### δ-Secretase cleaves SET and induces AD pathology

We also identified SET as a substrate of δ-secretase. SET is a multifunctional protein. It is an inhibitor of both DNase and protein phosphatase 2A (PP2A). In the AD brain, δ-secretase translocates from the lysosome to the cytoplasm or the nucleus and cleaves SET [3, 51]. The cleaved SET fragments lose their DNase inhibitor activity, inducing genomic DNA nicking and cell death in neurons [3]. Furthermore, SET is an inhibitor of protein phosphatase 2A (PP2A) and a regulator of tau phosphorylation [52]. The δ-secretase-derived SET fragments inhibit the activation of PP2A, triggering the hyperphosphorylation and aggregation of tau in AD, brain ischemia, and traumatic brain injury [51, 53, 54]. The overexpression of δ-secretase-derived SET fragments in the brain reproduces the key features of AD in rats [55], suggesting that the cleavage of SET is sufficient to induce AD-like pathology in rodent models.

### δ-Secretase in PD and other neurodegenerative diseases

PD is the second most common neurodegenerative disease after AD. It is characterized by the degeneration of dopaminergic neurons in the par compacta of substantia nigra and the deposition of α-synuclein. However, the molecular mechanisms underlying α-synuclein aggregation and dopaminergic neuronal death remain elusive [56]. We found that α-synuclein is also a substrate of δ-secretase. δ-Secretase cleaves α-synuclein after N103, generating an α-synuclein (1–103) fragment that is more prone to aggregate and exhibits a neurotoxic effect [57]. The injection of virus encoding human α-synuclein into mouse brain induces the aggregation of α-synuclein and the degeneration of the nigrostriatal pathway [58]. However, the toxic effect of α-synuclein overexpression is alleviated in δ-secretase knockout mice [57]. Furthermore, the α-synuclein (1–103) fragment binds monoamine oxidase-B (MAO-B) and stimulates its enzymatic activity [59]. MAO-B subsequently oxidizes dopamine into the highly oxidative and toxic metabolite DOPAL, which in turn stimulates δ-secretase activity. The pathological effect of α-synuclein (1–103) is diminished by depleting MAO-B, indicating that the δ-secretase-derived α-synuclein (1–103) fragment induces PD-like pathology through MAO-B [59].

TAR DNA-binding protein 43 (TDP-43) is another δ-secretase substrate involved in neurodegenerative diseases. It is the cardinal protein in the most common subtypes of frontotemporal dementia (FTD) and amyotrophic lateral sclerosis (ALS) [60]. TDP-43 is a nuclear protein involved in RNA splicing. It mislocalizes to the cytoplasm and aggregates into insoluble inclusion bodies in FTD/ALS [60]. δ-Secretase cleaves TDP-43 after N291 and N306, generating TDP-43 fragments that can be detected in postmortem FTD brain tissues. Several proteases have been reported to cleave TDP-43 and modulate its function, including calpain [61, 62], caspase [63], and δ-secretase [64]. Currently, the relative contribution of different proteases to the pathological function of TDP-43 remains unknown. It should be noted that δ-secretase is abundantly expressed in microglia, thus δ-secretase in microglia might also contribute to the pathogenesis of neurodegenerative diseases.

### Transcriptional regulation of δ-secretase

δ-Secretase cleaves key regulators of neurodegenerative diseases such as APP, tau, α-synuclein, SET, and TDP-43, and plays a pivotal role in the pathogenesis of AD, PD, and FTD/ALS. Interestingly, both the mRNA and protein levels of δ-secretase increase in an age-dependent manner [39, 46]. However, how the aging process regulates the expression and enzymatic activity of δ-secretase remains unknown. We searched for the transcriptional factors that regulate the expression of δ-secretase. A computational analysis of the δ-secretase putative transcription factor binding sequences revealed dozens of putative transcription factors. We focused on aging-related transcription factors and found that CCAAT/enhancer binding proteins (C/EBPβ) regulates the transcription of δ-secretase both in vitro and *in vivo* [65]. The C/EBP family regulates the expression of genes critical to glial activation and neuroinflammation [66] and is implicated in neurodegenerative diseases and brain injury [67]. The deletion of C/EBPβ protects mice from neuronal cell death induced by excitotoxicity and ischemia [68]. The expression of C/EBPβ is increased during aging and in AD mouse models [65]. The overexpression of C/EBPβ in an AD mouse model accelerates the onset of AD-like pathogenesis and worsens cognitive dysfunctions, while a reduction in C/EBPβ expression decreases the expression of δ-secretase, APP and tau and reverses both amyloid and tau pathology and cognitive impairments. These results indicate that C/EBPβ might promote not only the expression of δ-secretase but also the expression of APP and tau [65].

BDNF is the most important neurotrophic factor in the brain. It binds and activates TrkB receptors, activating neurotrophic signals. BDNF/TrkB signaling regulates neuronal development, differentiation, and survival. It has been reported that BDNF/TrkB signaling is impaired in AD [69, 70]. We found that a deficiency in BDNF/TrkB signaling activates the JAK2/STAT3 pathway, resulting in the upregulation of C/EBPβ, which is the transcriptional factor of δ-secretase [71]. Indeed, the blockade of BDNF/TrkB signaling increased δ-secretase expression and fragmentation of APP and tau, and promoted the onset of AD pathology in a mouse model of AD. The effect of BDNF/TrkB impairment was partially reversed by the knockdown of C/EBPβ. Further, the overexpression of an enzymatic-dead AEP C189S mutant also blocked the toxic effects of deficiency in the BDNF/TrkB signaling pathway. These results indicate that BDNF/TrkB reduction induces the overexpression of δ-secretase and AD-like pathology through upregulating C/EBPβ and δ-secretase expression [71].

In addition to BDNF/TrkB and C/EBPβ, other transcriptional factors are reported to regulate δ-secretase expression. For example, p53 binds to intron 1 of the human δ-secretase gene in a human colon cancer cell line [72]. Further study found that DJ-1, a protein implicated in cancer and PD, also binds to the p53 binding site of the mouse δ-secretase gene and regulates its expression. DJ-1 knockout increased the expression level of δ-secretase. The cleavage of the δ-secretase substrate annexin A2 was enhanced in DJ-1 knockout cells [73]. These results suggest that the DJ-1/p53/δ-secretase pathway may play a role in the pathogenesis of PD and cancers. Furthermore, it has been reported that nuclear calcium signals regulate the expression of δ-secretase [74]. It is still unknown whether calcium-regulated δ-secretase expression plays a role in neurodegenerative diseases.

### Posttranscriptional regulation of δ-secretase

δ-Secretase is synthesized as inactive prolegumain, which undergoes activation after self-cleavage of its N-terminal and C-terminal fragments under acidic conditions [11–14]. The maturation of δ-secretase is mainly regulated by the pH of the cell compartments. In vitro experiments found that the activity of purified legumain peaks at pH 5.5 [75]. Considering that the environments tend to be slightly acidic in cancer and AD [76–78], δ-secretase will be activated under these pathological conditions. Furthermore, the enzymatic activity of δ-secretase is also regulated by other factors. For example, in the presence of αVβ3 integrin, the pH optimum shifts from pH 5.5 to 6.0 [14], where αVβ3 integrin binds and stabilizes δ-secretase [14]. The glycosylation of prolegumain has been reported to be necessary for its transformation to an enzymatically active form [79]. Furthermore, naturally occurring polyanionic glycosaminoglycans (GAGs) accelerate the activation of prolegumain [80]. The ubiquitination of δ-secretase by TRAF6 at K63 promotes its intracellular stability as well as its secretion [81].

The cystatin family comprises inhibitors of cysteine protease, including δ-secretase. Cystatin E is the most potent endogenous δ-secretase inhibitor. Cystatin E is a secreted protein but is reported to be internalized into melanoma cells and regulate δ-secretase activity [82]. Cystatin E forms a domain-swapped dimer with increased conformational stability and forms a trimeric complex with δ-secretase. Cystatin E may further aggregate into amyloid fibrils, which still binds δ-secretase and blocks its enzymatic activity. However, the aggregated form of cystatin E may also stabilize δ-secretase, which can explain the existence of active δ-secretase at the near-neutral pH of the cytosol [83].

We studied the posttranslational modification of δ-secretase in neurons and found that Akt phosphorylates δ-secretase on residue T322. The phosphorylation of δ-secretase by Akt inhibits its autocleavage and enzymatic activation. The overexpression of the phosphor-mimetic δ-secretase T322E mutation in the tau P301S mouse brain inhibited δ-secretase activity, attenuated the cleavage of tau by δ-secretase, and alleviated the pathological and cognitive defects. AKT activation is regulated by the BDNF/TrkB signaling pathway. BDNF reduction attenuates δ-secretase phosphorylation on the T322 residue, eliciting δ-secretase cytosolic residency and activation [84]. Indeed, the treatment of an AD mouse model with a TrkB agonist inhibited the activation of δ-secretase and the pathological cleavage of APP and tau [85]. We also found that another kinase, serine-arginine protein kinase 2 (SRPK2), phosphorylates δ-secretase at the S226 residue. The phosphorylation of δ-secretase S226 is tightly correlated with SRPK2 activity in AD brains and promotes its autocatalytic cleavage, leading to escalated enzymatic activities. The phosphorylation mimetic mutation (S226D) of δ-secretase promotes AD pathology, while the nonphosphorylatable mutant (S226A) attenuates AD pathologies. Thus, the balance between T322 and S226 phosphorylation regulates the activity of δ-secretase in the brain [86].

### Targeting δ-secretase for the treatment of neurodegenerative diseases

Since δ-secretase is involved in the pathogenesis of neurodegenerative diseases, pharmacological agents inhibiting δ-secretase may be powerful therapeutic tools for treating many of these neurological diseases. Initially, most studies focus on Michael acceptor inhibitors. In 2002, a series of Michael acceptor inhibitors based on the backbone benzyloxycarbonyl-L-Ala-L-Ala-L-Asn was found to irreversibly inhibit mammalian δ-secretase [87]. Another group used alternative P2-P3 linker units and the P3 group SAR to identify Michael acceptor inhibitors and identified a cellularly active inhibitor that affects the cell viability and colony formation of cancer cells [88]. The in vivo pharmacokinetic profiling of these peptidyl δ-secretase inhibitors has not been tested. It remains unknown whether these peptidyl inhibitors have any appropriate druggability to treat neurodegenerative diseases. Generally, due to the intrinsic shortcomings, the peptidyl compounds possess poor pharmacokinetic profiles [89].

To develop small-molecule inhibitors of δ-secretase, we performed a high-throughput screen of 54,384 compounds with mouse kidney lysates and then counterscreened with kidney lysates from δ-secretase knockout mice [90]. A total of 736 hits from the preliminary screen were further screened with purified δ-secretase and identified 46 hits, which can be sorted into eight distinct substructure families. The most potent compounds from each group were tested with purified δ-secretase, and the IC_50_ values for the top 8 candidates were within the low micromolar range. Most of these compounds selectively inhibit δ-secretase but not other cysteine proteases, such as cathepsins and caspases. We further performed in vitro ADME profiles and analyzed the toxicity of the lead compounds. Only one compound (compound 11) passed the subsequent druggability evaluation. It is highly permeable in the Caco-2 monolayer permeability and BBB-PAMPA permeability assay, and it is relatively stable in the human liver microsomal stability assay. Furthermore, compound 11 does not affect cytochrome P450 enzymatic activity or cell viability. The kinetic analysis showed a biphasic inhibition mode of compound 11. Cocrystal structure analysis revealed a dual active site-directed and allosteric inhibition mode of this compound class. Compound 11 binds to the allosteric inhibition site flanking the b6 strand, located 30 Å away from the active site. It also bound to the active site of δ-secretase. We tested the effect of compound 11 in tau P301S and 5XFAD transgenic mouse models. Compound 11 was detected in the serum and brain tissues of mice, indicating that it penetrates the blood-brain barrier. Compound 11 inhibited the δ-secretase enzymatic activity, reduced the concentration of δ-secretase-cleaved tau and APP fragments, ameliorated synaptic loss and electrophysiological dysfunction, and reversed cognitive deficits [90]. This novel small-molecular compound may be an effective clinical therapeutic agent for AD and other neurodegenerative diseases. Since AEP knockout mice develop disorders of the hematopoietic system and kidney dysfunction [19, 24], special attention should be paid to the potential side effects of AEP inhibitors. On the other hand, it should be noted that the phenotypes induced by the knockout of a gene in animals do not mean necessarily that similar side-effects will occur upon inhibition of this gene by a small inhibitor. This is because knockout of a gene results in a complete eradication of the protein it encodes, whereas pharmacological inhibition only decreases its enzymatic function. The protein is still expressed and can potentially fulfil some other functions. Knockout is an extreme case, whereas inhibition merely antagonizes part of the enzymatic activities. Moreover, there are no such potent inhibitor that can inhibit 100% of the enzymatic effect. Hence, inhibition by the inhibitor does not necessarily causes the knockout-elicited side effects. Accordingly, we did not see any hematopoietic effects or kidney disorders in mice chronically exposed to compound 11. These results indicate that the partial inhibition of AEP in adult mice does not induce the phenotype in AEP knockout mice whose AEP is completely deleted during development [90]. The optimization of this lead compound is being actively pursued in our laboratory.

### δ-Secretase-generated fragments as diagnostic markers of AD

Despite the unprecedented advances in the field of AD research, the diagnosis of AD mainly depends on the clinical manifestations. Thus, the diagnosis is often missed or delayed in clinical practice. The potential pathology of AD begins many years before the appearance of cognitive impairment [91]. Methods to detect the presence of the underlying AD pathology would provide opportunities for early intervention to delay progressive cognitive decline. The well-recognized biomarkers in cerebrospinal fluid (CSF) include total tau (T-tau), phospho-181-Tau (P-Tau) and amyloid-β [92]. Several positron emission tomography (PET) tracers have been developed to detect the deposition of Aβ and tau in the brain [93]. These markers are sensitive and specific for the diagnosis of clinical AD, but their value in the diagnosis of prodromal AD is not well established.

Since δ-secretase cleaves APP and tau, initiating Aβ and tau pathology in the early stage of AD, the appearance of δ-secretase-related fragments may be a better biomarker for the diagnosis of AD. A study examined the link between CSF tau (1–368) levels and PET biomarkers [94]. The δ-secretase-derived tau (1–368) level in the CSF is associated with the distribution volume ratio (DVR) of the PET tau tracer [^18^F]THK5317 in the lateral temporal and frontal regions. The ratios between tau (1–368) and mid-domain tau are significantly associated with the [^18^F]THK5317 DVR in the temporal ROIs as well as in the ROIs of the parietal and occipital cortices. The tau-368/T-tau ratio and tau-368/tau N-Mid ratio are associated with lateral temporal and isocortical composite metabolism, respectively. CSF Tau-368 levels show a stronger association than T-tau and P-tau measures with regional [^18^F]THK5317 DVR. A longitudinal study found that the tau-368/tau N-Mid ratios are associated with the rate of change in Braak regions. The rates of change in [^18^F] FDG SUVR in the lateral temporal and posterior cingulate regions are significantly associated with the tau-368/T-tau ratio [94]. Clearly, more longitudinal studies are needed to confirm the predictive value of δ-secretase-generated tau, APP and other fragments for the early diagnosis of AD and other neurodegenerative diseases.

## Conclusions

**Fig. 1:**
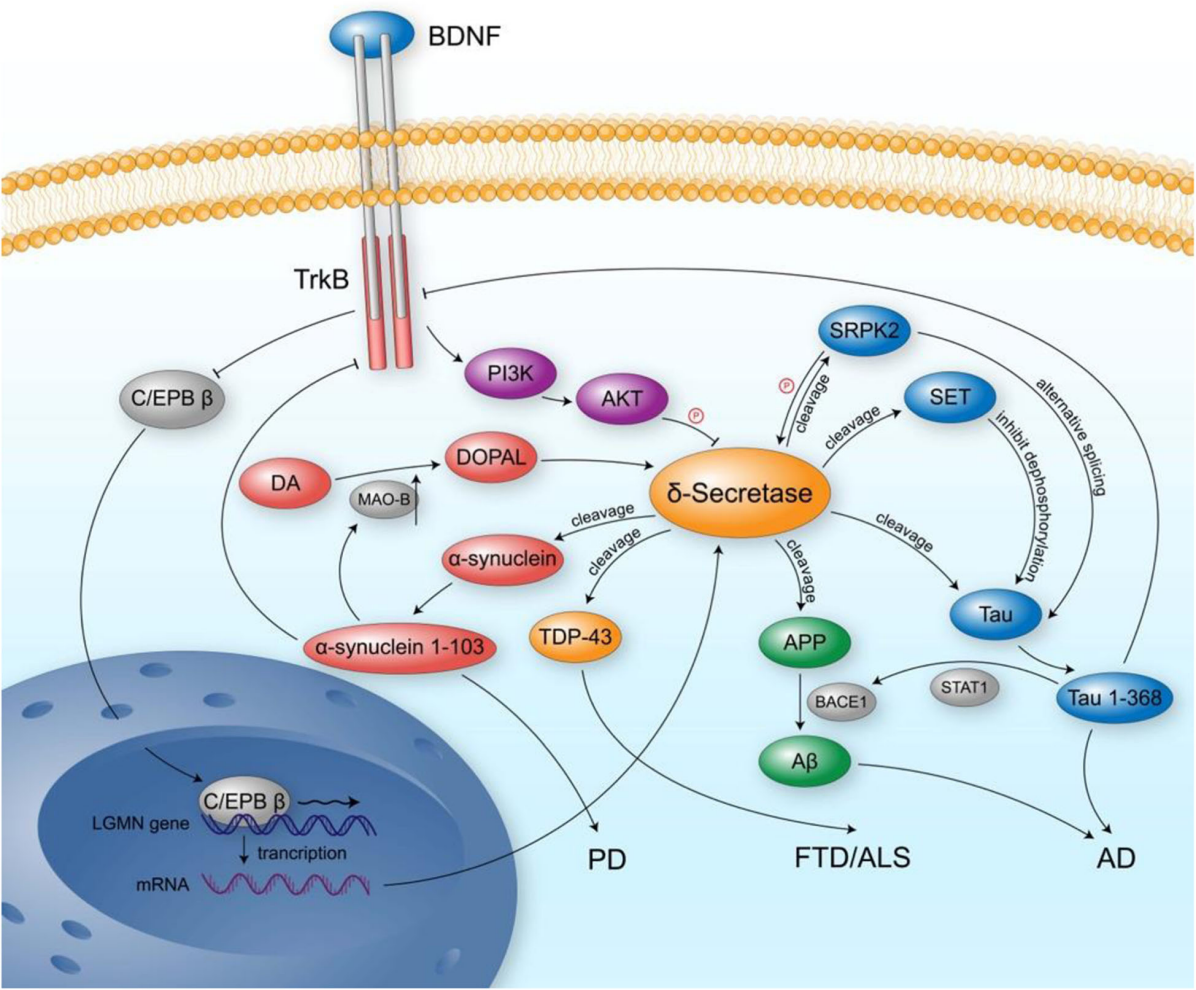
The regulation of δ-secretase in neurodegenerative diseases. The expression of δ-secretase is regulated by the transcriptional factor C/EBPβ, which is regulated by the BDNF/TrkB signalling pathway. δ-secretase is phosphorylated by Akt which inhibits its activity, and SRPK2 which enhances its activity. δ-secretase also cleaves α-synuclein, generating α-synuclein (1–103) fragment and promotes the onset of PD. Furthermore, α-synuclein (1–103) fragment activates MAO-B, which catalyses the oxidative deamination of dopamine and generation of DOPEL. DOPEL further activates δ-secretase. δ-secretase also cleaves TDP-43, which is indicated in FTD/ALS. δ-secretase cleaves APP, promoting the generation of Aβ. δ-secretase also cleaves tau, generating an aggregation-prone fragment. δ-secretase-derived tau fragment enhances BACE1 activity, which further facilitate Aβ production. δ-secretase-derived SET fragments inhibit the dephosphorylation of tau, while δ-secretase-derived SRPK2 fragment affects the alternative splicing of tau. All these pathways promote tau aggregation and the onset of AD

δ-Secretase is a novel protease that plays a pivotal role in the pathogenesis of neurodegenerative diseases. It cleaves its substrates such as tau, APP, SET, α-synuclein, SRPK2, and TDP-43, promoting the aggregation of Aβ, tau and α-synuclein, inducing neuronal cell death. δ-Secretase is tightly regulated at the transcriptional and posttranslational levels. C/EBPβ, an inflammatory cytokine-activated transcription factor, is the major transcriptional factor that regulates δ-secretase expression. Notably, the expression of C/EBPβ is regulated by the BDNF/TrkB signaling pathway. On the other hand, BDNF/TrkB also activates AKT, which phosphorylates δ-secretase at T322 and inhibits its enzymatic activity. The δ-secretase-cleaved tau (1–368) fragment in turn blocks the BDNF/TrkB signaling pathway. δ-Secretase is also phosphorylated by SRPK2 at S226, which promotes its protease activity (Fig. 1). Other posttranslational modifications, such as glycosylation and ubiquitination, also affect δ-secretase activity. Abundant evidence supports that δ-secretase may play a role in neurodegenerative disease. However, clinical evidence that can establish a definitive causal link between δ-secretase and neurodegenerative diseases is still lacking. It is believed that δ-secretase-modulated molecular targets may serve as diagnostic markers and therapeutic targets for AD. A series of Michael acceptor inhibitors, as well as small molecule inhibitors of legumain have been developed. The small molecule inhibitor compound 11 alleviates AD pathology in two mouse AD models. Because δ-secretase simultaneously cleaves both APP and Tau, it provides an unprecedent innovative target for treating this devastating neurodegenerative disease. Conceivably, further understanding of the biological and pathological mechanisms of δ-secretase will provide more novel diagnostic and therapeutic targets for neurodegenerative diseases.

## Data Availability

Not applicable.

## Abbreviations

AD: Alzheimer’s disease
AEP: Asparagine endopeptidase
ALS: Amyotrophic lateral sclerosis
APP: Amyloid precursor protein
Aβ: β-amyloid
BDNF: Brain-derived neurotrophic factor
C/EBPβ: CCAAT/ enhancer binding proteins
CSF: Cerebrospinal fluid
DVR: Distribution volume ratio
FTD: Frontotemporal dementia
GAGs: Glycosaminoglycans
PD: Parkinson’s disease
PET: Positron emission tomography
PP2A: Protein phosphatase 2A
PTC: Proximal tubular cells
SARs: Structure-activity relationships
SRPK2: Polyanionic serine-arginine protein kinase 2
TDP-43: TAR DNA-binding protein 43
TTCF: Tetanus toxin C fragment
VPE: Vacuolar processing enzyme

## References

[1] Collinge J (2016). Mammalian prions and their wider relevance in neurodegenerative diseases. Nature..

[2] Fu H, Hardy J, Duff KE (2018). Selective vulnerability in neurodegenerative diseases. Nat Neurosci.

[3] Liu Z, Jang SW, Liu X, Cheng D, Peng J, Yepes M (2008). Neuroprotective actions of PIKE-L by inhibition of SET proteolytic degradation by asparagine endopeptidase. Mol Cell.

[4] Dall E, Brandstetter H (2016). Structure and function of legumain in health and disease. Biochimie..

[5] Hara-Nishimura I, Takeuchi Y, Nishimura M (1993). Molecular characterization of a vacuolar processing enzyme related to a putative cysteine proteinase of Schistosoma mansoni. Plant Cell.

[6] Wu B, Yin J, Texier C, Roussel M, Tan KS (2010). Blastocystis legumain is localized on the cell surface, and specific inhibition of its activity implicates a pro-survival role for the enzyme. J Biol Chem.

[7] Dalton JP, Hola-Jamriska L, Brindley PJ (1995). Asparaginyl endopeptidase activity in adult Schistosoma mansoni. Parasitology.

[8] Hara-Nishimura I, Inoue K, Nishimura M (1991). A unique vacuolar processing enzyme responsible for conversion of several proprotein precursors into the mature forms. FEBS Lett.

[9] Vorster BJ, Cullis CA, Kunert KJ (2019). Plant vacuolar processing enzymes. Front Plant Sci.

[10] Hatsugai N, Kuroyanagi M, Yamada K, Meshi T, Tsuda S, Kondo M (2004). A plant vacuolar protease, VPE, mediates virus-induced hypersensitive cell death. Science..

[11] Li DN, Matthews SP, Antoniou AN, Mazzeo D, Watts C (2003). Multistep autoactivation of asparaginyl endopeptidase in vitro and in vivo. J Biol Chem.

[12] Dall E, Brandstetter H (2012). Activation of legumain involves proteolytic and conformational events, resulting in a context- and substrate-dependent activity profile. Acta Crystallogr Sect F Struct Biol Cryst Commun.

[13] Zhao L, Hua T, Crowley C, Ru H, Ni X, Shaw N (2014). Structural analysis of asparaginyl endopeptidase reveals the activation mechanism and a reversible intermediate maturation stage. Cell Res.

[14] Dall E, Brandstetter H (2013). Mechanistic and structural studies on legumain explain its zymogenicity, distinct activation pathways, and regulation. Proc Natl Acad Sci U S A.

[15] Dall E, Fegg JC, Briza P, Brandstetter H (2015). Structure and mechanism of an aspartimide-dependent peptide ligase in human legumain. Angew Chem Int Ed Engl.

[16] Jackson MA, Gilding EK, Shafee T, Harris KS, Kaas Q, Poon S (2018). Molecular basis for the production of cyclic peptides by plant asparaginyl endopeptidases. Nat Commun.

[17] Chen JM, Dando PM, Stevens RA, Fortunato M, Barrett AJ (1998). Cloning and expression of mouse legumain, a lysosomal endopeptidase. Biochem J.

[18] Morita Y, Araki H, Sugimoto T, Takeuchi K, Yamane T, Maeda T (2007). Legumain/asparaginyl endopeptidase controls extracellular matrix remodeling through the degradation of fibronectin in mouse renal proximal tubular cells. FEBS Lett.

[19] Miller G, Matthews SP, Reinheckel T, Fleming S, Watts C (2011). Asparagine endopeptidase is required for normal kidney physiology and homeostasis. FASEB J.

[20] Manoury B, Hewitt EW, Morrice N, Dando PM, Barrett AJ, Watts C (1998). An asparaginyl endopeptidase processes a microbial antigen for class II MHC presentation. Nature..

[21] Matthews SP, Werber I, Deussing J, Peters C, Reinheckel T, Watts C (2010). Distinct protease requirements for antigen presentation in vitro and in vivo. J Immunol.

[22] Watts C, Moss CX, Mazzeo D, West MA, Matthews SP, Li DN (2003). Creation versus destruction of T cell epitopes in the class II MHC pathway. Ann N Y Acad Sci.

[23] Manoury B, Mazzeo D, Li DN, Billson J, Loak K, Benaroch P (2003). Asparagine endopeptidase can initiate the removal of the MHC class II invariant chain chaperone. Immunity.

[24] Chan CB, Abe M, Hashimoto N, Hao C, Williams IR, Liu X (2009). Mice lacking asparaginyl endopeptidase develop disorders resembling hemophagocytic syndrome. Proc Natl Acad Sci U S A.

[25] Choi SJ, Reddy SV, Devlin RD, Menaa C, Chung H, Boyce BF (1999). Identification of human asparaginyl endopeptidase (legumain) as an inhibitor of osteoclast formation and bone resorption. J Biol Chem.

[26] Choi SJ, Kurihara N, Oba Y, Roodman GD (2001). Osteoclast inhibitory peptide 2 inhibits osteoclast formation via its C-terminal fragment. J Bone Miner Res.

[27] Hardy JA, Higgins GA (1992). Alzheimer’s disease: the amyloid cascade hypothesis. Science..

[28] Beyreuther K, Masters CL (1991). Amyloid precursor protein (APP) and beta A4 amyloid in the etiology of Alzheimer’s disease: precursor-product relationships in the derangement of neuronal function. Brain Pathol.

[29] Selkoe DJ (1991). The molecular pathology of Alzheimer’s disease. Neuron..

[30] Ittner LM, Ke YD, Delerue F, Bi M, Gladbach A, van Eersel J (2010). Dendritic function of tau mediates amyloid-beta toxicity in Alzheimer's disease mouse models. Cell..

[31] Rapoport M, Dawson HN, Binder L, Vitek MP, Ferreira A (2002). Tau is essential to beta-amyloid-induced neurotoxicity. Proc Natl Acad Sci U S A.

[32] Egan MF, Kost J, Voss T, Mukai Y, Aisen PS, Cummings JL (2019). Randomized trial of Verubecestat for prodromal Alzheimer's disease. N Engl J Med.

[33] Egan MF, Kost J, Tariot PN, Aisen PS, Cummings JL, Vellas B (2018). Randomized trial of Verubecestat for mild-to-moderate Alzheimer's disease. N Engl J Med.

[34] Honig LS, Vellas B, Woodward M, Boada M, Bullock R, Borrie M (2018). Trial of Solanezumab for mild dementia due to Alzheimer's disease. N Engl J Med.

[35] Salloway S, Sperling R, Fox NC, Blennow K, Klunk W, Raskind M (2014). Two phase 3 trials of bapineuzumab in mild-to-moderate Alzheimer's disease. N Engl J Med.

[36] Müller UC, Deller T, Korte M (2017). Not just amyloid: physiological functions of the amyloid precursor protein family. Nat Rev Neurosci.

[37] Gervais FG, Xu D, Robertson GS, Vaillancourt JP, Zhu Y, Huang J (1999). Involvement of caspases in proteolytic cleavage of Alzheimer's amyloid-beta precursor protein and amyloidogenic a beta peptide formation. Cell..

[38] Willem M, Tahirovic S, Busche MA, Ovsepian SV, Chafai M, Kootar S (2015). η-Secretase processing of APP inhibits neuronal activity in the hippocampus. Nature..

[39] Zhang Z, Song M, Liu X, Su Kang S, Duong DM, Seyfried NT (2015). Delta-secretase cleaves amyloid precursor protein and regulates the pathogenesis in Alzheimer’s disease. Nat Commun.

[40] Zhao X, Kotilinek LA, Smith B, Hlynialuk C, Zahs K, Ramsden M (2016). Caspase-2 cleavage of tau reversibly impairs memory. Nat Med.

[41] de Calignon A, Fox LM, Pitstick R, Carlson GA, Bacskai BJ, Spires-Jones TL (2010). Caspase activation precedes and leads to tangles. Nature..

[42] Rissman RA, Poon WW, Blurton-Jones M, Oddo S, Torp R, Vitek MP (2004). Caspase-cleavage of tau is an early event in Alzheimer disease tangle pathology. J Clin Invest.

[43] Park SY, Ferreira A (2005). The generation of a 17 kDa neurotoxic fragment: an alternative mechanism by which tau mediates beta-amyloid-induced neurodegeneration. J Neurosci.

[44] Chen HH, Liu P, Auger P, Lee SH, Adolfsson O, Rey-Bellet L (2018). Calpain-mediated tau fragmentation is altered in Alzheimer's disease progression. Sci Rep.

[45] Arai T, Guo JP, McGeer PL (2005). Proteolysis of non-phosphorylated and phosphorylated tau by thrombin. J Biol Chem.

[46] Zhang Z, Song M, Liu X, Kang SS, Kwon IS, Duong DM (2014). Cleavage of tau by asparagine endopeptidase mediates the neurofibrillary pathology in Alzheimer's disease. Nat Med.

[47] Xiang J, Wang ZH, Ahn EH, Liu X, Yu SP, Manfredsson FP (2019). Delta-secretase-cleaved tau antagonizes TrkB neurotrophic signalings, mediating Alzheimer’s disease pathologies. Proc Natl Acad Sci U S A.

[48] Hong Y, Chan CB, Kwon IS, Li X, Song M, Lee HP (2012). SRPK2 phosphorylates tau and mediates the cognitive defects in Alzheimer's disease. J Neurosci.

[49] Wang ZH, Liu P, Liu X, Yu SP, Wang JZ, Ye K (2018). Delta-secretase (AEP) mediates tau-splicing imbalance and accelerates cognitive decline in tauopathies. J Exp Med.

[50] Zhang Z, Li XG, Wang ZH, Song M, Yu SP, Kang SS, et al. δ-Secretase-cleaved tau stimulates Aβ production via upregulating STAT1-BACE1 signaling in Alzheimer’s disease. Mol Psychiatry. 2018. 10.1038/s41380-018-0286-z. 10.1038/s41380-018-0286-z PMC6684859 30382187

[51] Basurto-Islas G, Grundke-Iqbal I, Tung YC, Liu F, Iqbal K (2013). Activation of asparaginyl endopeptidase leads to tau hyperphosphorylation in Alzheimer disease. J Biol Chem.

[52] Liu C, Götz J (2013). How it all started: tau and protein phosphatase 2A. J Alzheimers Dis.

[53] Basurto-Islas G, Gu JH, Tung YC, Liu F, Iqbal K (2018). Mechanism of tau hyperphosphorylation involving lysosomal enzyme asparagine endopeptidase in a mouse model of brain ischemia. J Alzheimers Dis.

[54] Hu W, Tung YC, Zhang Y, Liu F, Iqbal K (2018). Involvement of activation of Asparaginyl Endopeptidase in tau hyperphosphorylation in repetitive mild traumatic brain injury. J Alzheimers Dis.

[55] Bolognin S, Blanchard J, Wang X, Basurto-Islas G, Tung YC, Kohlbrenner E (2012). An experimental rat model of sporadic Alzheimer’s disease and rescue of cognitive impairment with a neurotrophic peptide. Acta Neuropathol.

[56] Poewe W, Seppi K, Tanner CM, Halliday GM, Brundin P, Volkmann J (2017). Parkinson disease. Nat Rev Dis Primers.

[57] Zhang Z, Kang SS, Liu X, Ahn EH, Zhang Z, He L (2017). Asparagine endopeptidase cleaves α-synuclein and mediates pathologic activities in Parkinson's disease. Nat Struct Mol Biol.

[58] Koprich JB, Kalia LV, Brotchie JM (2017). Animal models of α-synucleinopathy for Parkinson disease drug development. Nat Rev Neurosci.

[59] Kang SS, Ahn EH, Zhang Z, Liu X, Manfredsson FP, Sandoval IM (2018). α-Synuclein stimulation of monoamine oxidase-B and legumain protease mediates the pathology of Parkinson’s disease. EMBO J.

[60] Mackenzie IR, Rademakers R, Neumann M (2010). TDP-43 and FUS in amyotrophic lateral sclerosis and frontotemporal dementia. Lancet Neurol.

[61] Yamashita T, Hideyama T, Hachiga K, Teramoto S, Takano J, Iwata N (2012). A role for calpain-dependent cleavage of TDP-43 in amyotrophic lateral sclerosis pathology. Nat Commun.

[62] Rao MV, Campbell J, Palaniappan A, Kumar A, Nixon RA (2016). Calpastatin inhibits motor neuron death and increases survival of hSOD1(G93A) mice. J Neurochem.

[63] Zhang YJ, Xu YF, Cook C, Gendron TF, Roettges P, Link CD (2009). Aberrant cleavage of TDP-43 enhances aggregation and cellular toxicity. Proc Natl Acad Sci U S A.

[64] Herskowitz JH, Gozal YM, Duong DM, Dammer EB, Gearing M, Ye K (2012). Asparaginyl endopeptidase cleaves TDP-43 in brain. Proteomics..

[65] Wang ZH, Gong K, Liu X, Zhang Z, Sun X, Wei ZZ (2018). C/EBPβ regulates delta-secretase expression and mediates pathogenesis in mouse models of Alzheimer's disease. Nat Commun.

[66] Wang H, Liu X, Chen S, Ye K (2018). Spatiotemporal activation of the C/EBPβ/δ-secretase axis regulates the pathogenesis of Alzheimer's disease. Proc Natl Acad Sci U S A.

[67] Cortés-Canteli M, Wagner M, Ansorge W, Pérez-Castillo A (2004). Microarray analysis supports a role for ccaat/enhancer-binding protein-beta in brain injury. J Biol Chem.

[68] Cortes-Canteli M, Luna-Medina R, Sanz-Sancristobal M, Alvarez-Barrientos A, Santos A, Perez-Castillo A (2008). CCAAT/enhancer binding protein beta deficiency provides cerebral protection following excitotoxic injury. J Cell Sci.

[69] Ginsberg SD, Malek-Ahmadi MH, Alldred MJ, Chen Y, Chen K, Chao MV (2019). Brain-derived neurotrophic factor (BDNF) and TrkB hippocampal gene expression are putative predictors of neuritic plaque and neurofibrillary tangle pathology. Neurobiol Dis.

[70] Jerónimo-Santos A, Vaz SH, Parreira S, Rapaz-Lérias S, Caetano AP, Buée-Scherrer V (2015). Dysregulation of TrkB receptors and BDNF function by amyloid-β peptide is mediated by Calpain. Cereb Cortex.

[71] Wang ZH, Xiang J, Liu X, Yu SP, Manfredsson FP, Sandoval IM (2019). Deficiency in BDNF/TrkB Neurotrophic Activity Stimulates δ-Secretase by Upregulating C/EBPβ in Alzheimer’s Disease. Cell Rep.

[72] Yamane T, Murao S, Kato-Ose I, Kashima L, Yuguchi M, Kozuka M (2013). Transcriptional regulation of the legumain gene by p53 in HCT116 cells. Biochem Biophys Res Commun.

[73] Yamane T, Yamamoto Y, Nakano Y, Nakagaki T, Ohkubo I, Ariga H (2015). Expression and protease activity of mouse legumain are regulated by the oncogene/transcription co-activator, DJ-1 through p53 and cleavage of annexin A2 is increased in DJ-1-knockout cells. Biochem Biophys Res Commun.

[74] Andrade V, Guerra M, Jardim C, Melo F, Silva W, Ortega JM (2011). Nucleoplasmic calcium regulates cell proliferation through legumain. J Hepatol.

[75] Chen JM, Dando PM, Rawlings ND, Brown MA, Young NE, Stevens RA (1997). Cloning, isolation, and characterization of mammalian legumain, an asparaginyl endopeptidase. J Biol Chem.

[76] Yates CM, Butterworth J, Tennant MC, Gordon A (1990). Enzyme activities in relation to pH and lactate in postmortem brain in Alzheimer-type and other dementias. J Neurochem.

[77] Ji K, Mayernik L, Moin K, Sloane BF (2019). Acidosis and proteolysis in the tumor microenvironment. Cancer Metastasis Rev.

[78] Rohani N, Hao L, Alexis MS, Joughin BA, Krismer K, Moufarrej MN (2019). Acidification of tumor at stromal boundaries drives Transcriptome alterations associated with aggressive phenotypes. Cancer Res.

[79] Lunde NN, Haugen MH, Bodin Larsen KB, Damgaard I, Pettersen SJ, Kasem R (2017). Glycosylation is important for legumain localization and processing to active forms but not for cystatin E/M inhibitory functions. Biochimie..

[80] Berven L, Johansen HT, Solberg R, Kolset SO, Samuelsen AB (2013). Autoactivation of prolegumain is accelerated by glycosaminoglycans. Biochimie..

[81] Lin Y, Qiu Y, Xu C, Liu Q, Peng B, Kaufmann GF (2014). Functional role of asparaginyl endopeptidase ubiquitination by TRAF6 in tumor invasion and metastasis. J Natl Cancer Inst.

[82] Wallin H, Apelqvist J, Andersson F, Ekström U, Abrahamson M (2017). Low-level internalization of cystatin E/M affects legumain activity and migration of melanoma cells. J Biol Chem.

[83] Dall E, Hollerweger JC, Dahms SO, Cui H, Häussermann K, Brandstetter H (2018). Structural and functional analysis of cystatin E reveals enzymologically relevant dimer and amyloid fibril states. J Biol Chem.

[84] Wang ZH, Wu W, Kang SS, Liu X, Wu Z, Peng J (2018). BDNF inhibits neurodegenerative disease-associated asparaginyl endopeptidase activity via phosphorylation by AKT. JCI insight.

[85] Chen C, Wang Z, Zhang Z, Liu X, Kang SS, Zhang Y (2018). The prodrug of 7,8-dihydroxyflavone development and therapeutic efficacy for treating Alzheimer’s disease. Proc Natl Acad Sci U S A.

[86] Wang ZH, Liu P, Liu X, Manfredsson FP, Sandoval IM, Yu SP (2017). Delta-Secretase Phosphorylation by SRPK2 Enhances Its Enzymatic Activity, Provoking Pathogenesis in Alzheimer’s Disease. Mol Cell.

[87] Niestroj AJ, Feussner K, Heiser U, Dando PM, Barrett A, Gerhartz B (2002). Inhibition of mammalian legumain by Michael acceptors and AzaAsn-halomethylketones. Biol Chem.

[88] Ness KA, Eddie SL, Higgins CA, Templeman A, D'Costa Z, Gaddale KK (2015). Development of a potent and selective cell penetrant Legumain inhibitor. Bioorg Med Chem Lett.

[89] Mahato RI, Narang AS, Thoma L, Miller DD (2003). Emerging trends in oral delivery of peptide and protein drugs. Crit Rev Ther Drug Carrier Syst.

[90] Zhang Z, Obianyo O, Dall E, Du Y, Fu H, Liu X (2017). Inhibition of delta-secretase improves cognitive functions in mouse models of Alzheimer's disease. Nat Commun.

[91] Jack CR, Knopman DS, Jagust WJ, Petersen RC, Weiner MW, Aisen PS (2013). Tracking pathophysiological processes in Alzheimer's disease: an updated hypothetical model of dynamic biomarkers. Lancet Neurol.

[92] Banning LCP, Ramakers IHGB, Deckers K, Verhey FRJ, Aalten P (2019). Affective symptoms and AT(N) biomarkers in mild cognitive impairment and Alzheimer's disease: a systematic literature review. Neurosci Biobehav Rev.

[93] Villemagne VL, Dore V, Burnham SC, Masters CL, Rowe CC (2018). Imaging tau and amyloid-β proteinopathies in Alzheimer disease and other conditions. Nat Rev Neurol.

[94] Leuzy A, Cicognola C, Chiotis K, Saint-Aubert L, Lemoine L, Andreasen N (2019). Longitudinal tau and metabolic PET imaging in relation to novel CSF tau measures in Alzheimer’s disease. Eur J Nucl Med Mol Imaging.

